# Fabrication of Dielectric Elastomer Composites by Locking a Pre-Stretched Fibrous TPU Network in EVA

**DOI:** 10.3390/ma11091687

**Published:** 2018-09-12

**Authors:** Liang Jiang, Yanfen Zhou, Yuhao Wang, Zhiqing Jiang, Fang Zhou, Shaojuan Chen, Jianwei Ma

**Affiliations:** Department of Textile Materials and Textile Design, College of Textiles and Clothing, Qingdao University, Qingdao 266071, China; liang.jiang@outlook.com (L.J.); yuhao_wong@outlook.com (Y.W.); zhiqing.jiang@outlook.com (Z.J.); sdjnzhouf@163.com (F.Z.); mjwfz@hotmail.com (J.M.)

**Keywords:** thermoplastic polyurethane, dielectric elastomer, EVA, electromechanical properties

## Abstract

Dielectric elastomer (DE) composites with high electrical breakdown strength and large voltage-induced deformation were developed by retaining pre-stretched thermoplastic polyurethane (TPU) fibers in ethylene vinyl acetate copolymer (EVA). The microstructure of the candidate E-TPU fiber membrane and EVA coated E-TPU (E-TPU/EVA) film were characterized by scanning electron microscopy (SEM). The quasi-static and dynamic mechanical property, and the electromechanical properties, including the dielectric constant, dielectric loss tangent, and electromechanical sensitivity, of the DE composites were evaluated. Initially, tensile tests demonstrated that the DE composites based on E-TPU/EVAs had a higher elongation at break of above 1000% but a low elastic modulus of approximately 1.7 MPa. Furthermore, dielectric spectroscopy showed that the E-TPU/EVA had a dielectric constant of 4.5 at the frequency of 1000 Hz, which was 1.2 times higher than that of pure EVA film. Finally, it was found from electromechanical test that the voltage induced strain of E-TPU/EVA rose to 6%, nearly 3 times higher than that of pure TPU film, indicating an excellent electromechanical property. The DE composites developed have demonstrated the potential to be good candidate materials in the fields of artificial intelligence, biomimicry and renewable energy.

## 1. Introduction

**Figure 1:**
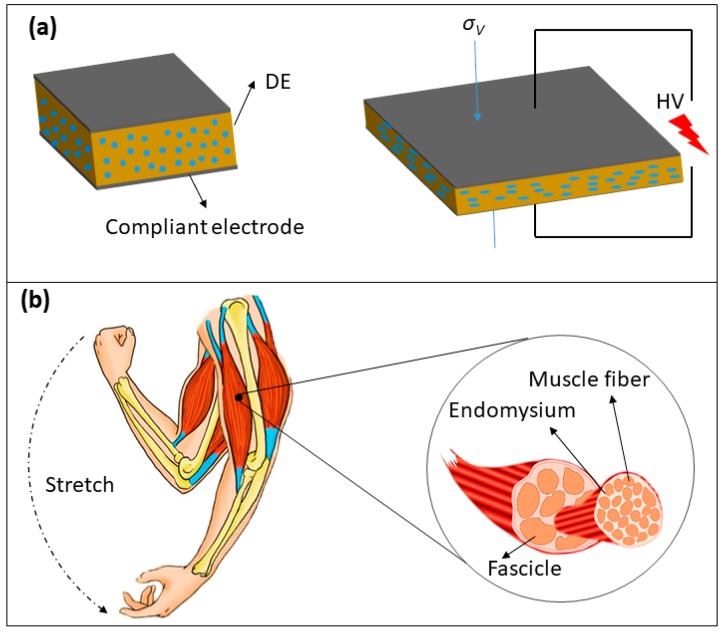
(**a**) The working principle of DEs; (**b**) the structure of human muscle.

Dielectric elastomers (DEs) are considered smart materials capable of responding to electrical stimuli by changing their shapes [1,2]. The working mechanism of DEs (shown in Figure 1a) can be described thus: the upper and bottom surfaces of the DE are coated with compliant electrodes (normally carbon-based materials); when a voltage is applied, an electrostatic force is produced between the upper and bottom surfaces of the DE, and this leads to a decrease in its thickness and a consequent increase in its area; when the voltage is off, the DE can rapidly recover its original shapes [3,4,5,6]. DEs are sometimes referred to as “artificial muscle” materials because they can readily resemble natural muscle under strain, as well as display similar actuation pressures, response speeds, electromechanical energy densities and coupling efficiencies. Consequently, they have been proposed for multiple applications in the fields of biomimicry, artificial intelligence, sensors and energy harvesting [7,8,9,10,11]. Generally, the actuated strain of DEs can be determined by using Equation (1) [12,13,14]:

(1) $$s_{z}=-\sigma_{V}/Y\;=-\varepsilon^{\prime }\varepsilon_{0}\varphi^{2}/Y\;$$

where *ε*′ is the relative permittivity or the dielectric constant of the DE material, *ε*_0_ is the permittivity of the free space (8.85 × 10^−12^ F/m), *Y* is the Young’s modulus of the DE material and *φ* is the electric field strength which equals the applied high voltage (*Φ*) divided by the thickness of the DE (*z*). According to Equation (1), the electromechanical performance of DEs can be improved by either increasing the dielectric constant or reducing the Young’s modulus (or a combination of both). Additionally, it has previously been reported that the electromechanical properties of DEs can be further increased by pre-stretching due to the decrease in thickness and an unpredicted increase in electrical breakdown strength [15,16,17]. However, pre-stretch was generally applied to DE films by a traditional peripheral unit which is normally large and heavy, leading to the reduction of electromechanical properties [18].

DE materials are usually made from acrylate [19,20,21,22], silicone rubber (SR) [23,24,25] or polyurethane (PU) [26,27]. Of these materials, PU has the highest dielectric constant which is a property beneficial for electromechanical performance. However, PU is much stiffer than the other two materials and has lower electrical resistivity than them, which significantly limits its application in DEs [27,28,29]. In recent years, electrospinning technology [30,31] has provided a versatile and efficient strategy for producing fluffy and soft nanofibers based membranes with high porosity [32].

In this study, a novel DE composite based on a micro/nano-structured TPU fibrous membrane (E-TPU) was prepared with the aim of achieving a large voltage-induced deformation by reducing the elastic modulus of the composite. The ethylene vinyl acetate (EVA) copolymer, which possesses flexibility, biocompatibility and high electrical breakdown strength, refs. [33,34,35] was coated onto and penetrated the pre-stretched E-TPU membrane. This was done to retain the pre-stretch of the TPU fibers and hence producing DEs with enhanced electromechanical performance. The resultant DE composites, having high dielectric constants, resembled fascicle structures akin to human muscles, with E-TPU fibers acting as muscle fibers and EVA as endomysium (see Figure 1b). The type of biomimicry structured in the E-TPU/EVA composites has rarely been reported on before.

## 2. Materials and Methods

### 2.1. Materials

A commercial TPU T3170, which has an elongation at break of 870%, was purchased from Shandong INOV New Materials Co., Ltd. (Zibo, Shandong, China). The chemical structure of TPU consisted of polyols and the short-chain diols reacted with the diisocyanates through polyaddition. The commercially available conductive carbon grease NYOGEL 756 G used as the compliant electrode in this work was provided by Nye Lubricants, Inc. (Fairhaven, MA, USA). EVA with a comonomer content of above 41% was purchased from the Arkema Company (Shanghai, China). *N*,*N*-dimethylformamide (DMF) and tetrahydrofuran (THF) were purchased from Sigma-Aldrich (Shanghai, China). All chemicals were used as received without further purification.

### 2.2. Preparation of E-TPU Membrane

Firstly, TPU pellets were dried in an electric vacuum oven at 80 °C for 2 h to remove moisture. Then, the pellets were dissolved in a mixture of 50 wt % DMF and 50 wt % THF with mass ratios of 8%, 10% and 12%, respectively. Afterwards, the solution was heated at 60 °C for 1 h under magnetic stirring to accelerate the dissolving. Stirring was continued for another 6 h without heating to ensure that complete dissolving was achieved. Prior to using, the solutions were left at room temperature overnight. To fabricate the E-TPU membrane, the solution was loaded in a plastic syringe connected to an 18 gauge blunt end needle and the syringe was mounted on a digital syringe pump from Longer Precision Pump Co., Ltd. (Baoding, Hebei, China). The electrospinning procedure was carried out using a voltage of 20 kV, a working distance of 20 cm between the capillary tip and the collector, and a flow rate of 1.2 mL/h. The resultant TPU membrane was left in a fume hood overnight at room temperature to remove the solvent prior to further processing.

### 2.3. Fabrication of E-TPU/EVA Composites and TPU Film

10 g EVA chips were dissolved in 40 g xylene with heating and magnetic stirring at 60 °C for 1 h and the mixture was thereafter stirred without heating for another 6 h. Then, it was applied as a coat to E-TPU membranes, which were pre-stretched by applying an equi-biaxial stretch ratio of 1.4 using an in-house pre-stretch rig, utilizing the blade coating method with a controlled thickness. The E-TPU/EVA composite film was obtained after air-drying for 24 h. For comparison, TPU film was also prepared with a TPU solution by using the doctoral blade coating method.

### 2.4. Characterizations

The microstructure of electrospun membranes with TPU concentrations of 8 wt %, 10 wt %, 12 wt % and E-TPU/EVA film were determined using a Scanning Electron Microscope (SEM) from Phenom Pro (Eindhoven, Netherlands). Samples were sputtered with a thin layer of gold before observation and images of different magnifications were taken at an accelerating voltage of 5 kV.

Tensile tests were performed using a Zwick Z010 tensile testing machine from Zwick Roell (Kennesaw, GA, USA) with a crosshead speed of 100 mm/min. Specimens with a gauge width of 10 mm and a gauge length of 18 mm were prepared through die cutting. Young’s modulus was determined from the slope of the stress–strain curve using a linear fit to the data points obtained within 5% strain. Three tests were conducted for each sample and the average value from each was used.

Dynamic mechanical analysis was carried out on a Q800 DMA from TA Instruments (New Castle, DE, USA) using the multi-frequency-strain mode. Strips of 7 mm width, 14 mm long, and 0.15 mm thickness were thermally equilibrated at −50 °C and heated to 150 °C at a rate of 3 °C/min while subjected to a 0.5% dynamic strain at 1 Hz.

Dielectric properties of the prepared DE composites were measured by using broad-band dielectric spectroscopy from Alpha-A Novocontrol (Montabaur, Germany) in the frequency range of 1 Hz to 10^6^ Hz at room temperature. The sample was placed on a cell which was a disposable gold-plated flat electrode with a diameter of 20 mm and thickness of 2 mm. During testing, the sample was just in contact with the fixture in the absence of pressure. The experimental data point of dielectric properties was the average of the results obtained from at least five samples under the same testing conditions.

**Figure 2:**
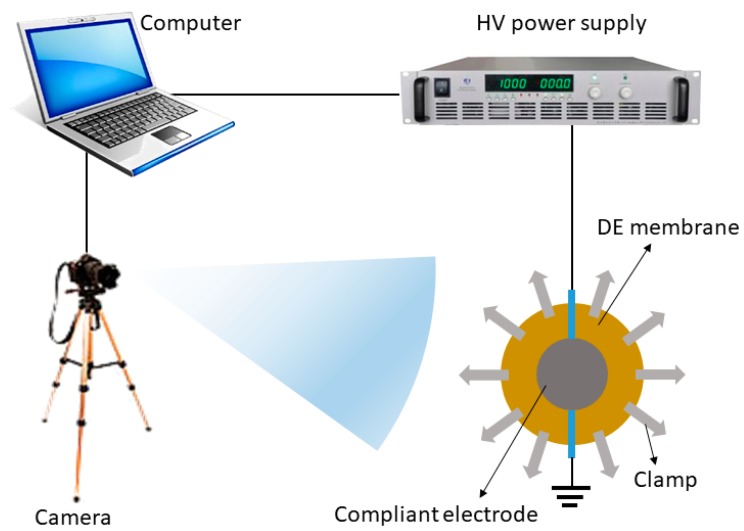
Schematic illustration of the electromechanical testing system.

The electromechanical testing system consisting of a camera, a test rig which was used to clamp samples and a high voltage power supply is shown in Figure 2. Samples were coated with the compliant electrode and clamped in the rig. The camera recorded the changes in area when an electric field was incrementally applied in voltage steps of 0.5 kV at 10 s interval. In order to measure the actuated area strain (*s_a_*), a camera recorded area changes of the samples for each increment of applied voltage. Thereafter, the initial area (*A*_0_) and the actuated area (*A*) were accurately measured using ImageJ software. The area strain *s_a_* was calculated from the following formula (Equation (2))

(2) $$\;s_{a}=\left(A-A_{0}\right)/A_{0}\;$$

## 3. Results and Discussion

**Figure 3:**
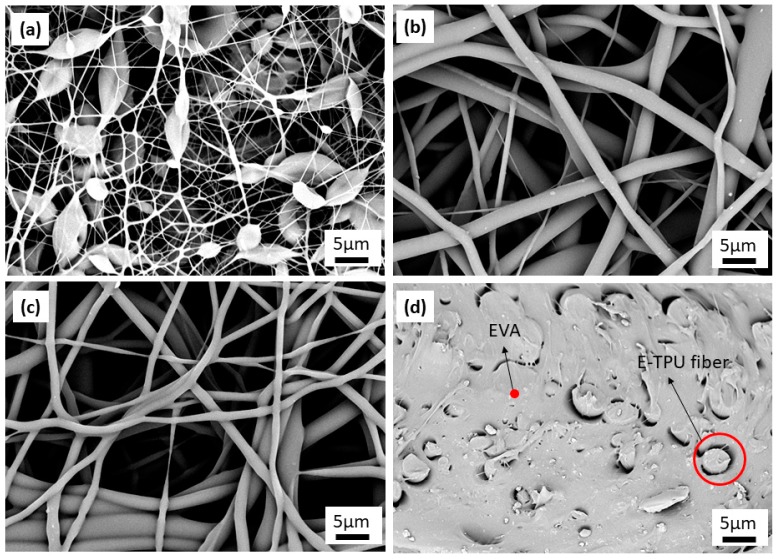
SEM images of TPU membranes with a mass ratio of (**a**) 8%, (**b**)10% and (**c**) 12% using a mixing solution comprising DMF and THF at a ratio of 1:1; (**d**) the cross-section image of an EVA coated E-TPU (E-TPU/EVA) membrane.

The microstructure of electrospun TPU membranes from TPU concentrations of 8 wt %, 10 wt % and 12 wt % of TPU together with E-TPU/EVA membrane are shown in Figure 3. It can be seen from Figure 3a that the fibers generated from the 8 wt % TPU solution showed a large number of beads. This was because the TPU solution was too dilute to be stretched into smooth fibers. However, the beads disappeared in the electrospun membranes with TPU concentrations of 10 wt % and 12 wt %. As can be observed in Figure 3b,c the electrospun membrane with the 10 wt % TPU solution showed a good morphology and had a chaotic fiber distribution with the largest fiber diameters being up to 3 μm, while the electrospun membrane with 12 wt % TPU solution presented a greater adhesion area than that of the electrospun membrane with 10 wt % TPU. Based on this, the electrospun TPU membrane with 10 wt % TPU solution was used to fabricate DE composites by coating a layer of EVA onto their surfaces. The cross-section of E-TPU membrane embedded EVA is presented in Figure 3d. It can be seen that the E-TPU/EVA composites appeared as ‘islands isolated in a sea’. However, the interface between TPU fibers and EVA was clearly discernable indicating poor compatibility between the two components. This problem needs to be addressed in future research.

**Figure 4:**
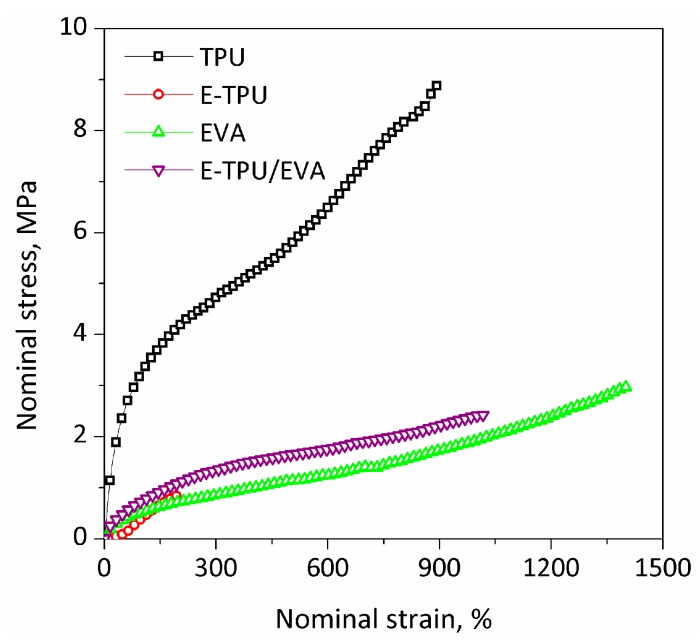
The dependence of nominal strain on nominal stress.

The quasi-static mechanical properties of TPU films, electrospun TPU (E-TPU) membranes, EVA films and E-TPU/EVA composites were evaluated and plots of nominal stress versus nominal strain are shown in Figure 4. The corresponding data are presented in Table 1. It can be seen that TPU film had an elongation at break of above 900% and a tensile strength of 8.9 MPa, while E-TPU fibers unsurprisingly exhibited an elongation at break of 200% and a tensile strength of 0.9 MPa. The weak mechanical properties of the electrospun membranes are caused by the highly porous structures, small fiber diameters and weak bonding between the nanofibers [36]. The elongation at break and tensile strength of EVA film was determined to be 1400% and 3.0 MPa, respectively. By coating E-TPU fibers with EVA, the tensile strength at break increased to 2.4 MPa which was 2 times higher than that of E-TPU fiber membrane. The maximum strain of the composite was more than 1000%, which was 5 times larger than that of E-TPU fiber membrane. The increase in both tensile strength and maximum strain was mainly because EVA formed a polymer network by penetrating into and subsequently reinforced the pre-stretched E-TPU.

**Figure 5:**
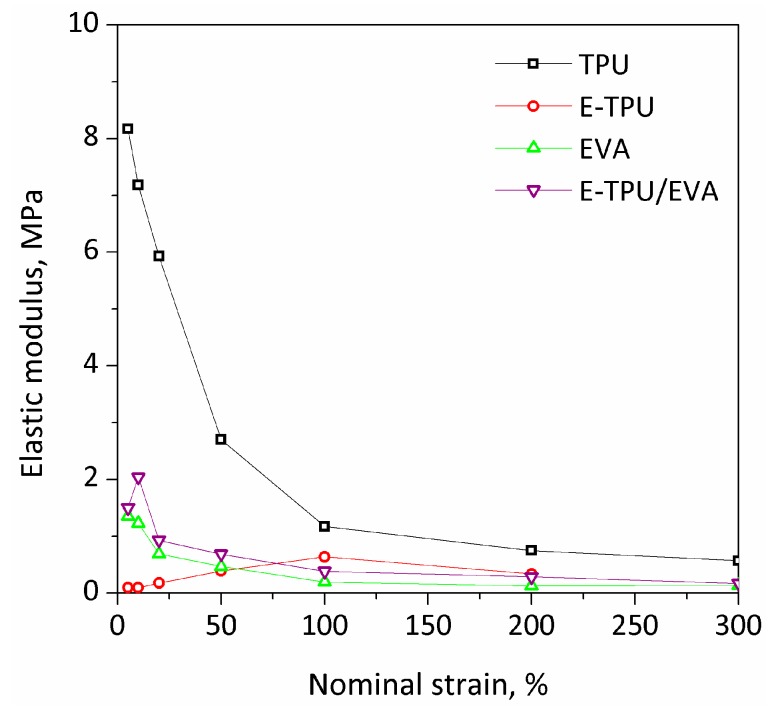
The relation of elastic modulus to nominal strain.

The relation between elastic modulus and nominal strain from 0% to 300% for TPU, E-TPU, EVA and E-TPU/EVA was determined and the results are presented in Figure 5. It can be noted that the TPU film had the highest elastic modulus over the full strain range. Furthermore, the elastic modulus of TPU film decreased significantly from 8.17 MPa to 0.75 MPa with the increase in strain, indicating the domination of strain softening in the whole strain range. A similar trend was found in the change of the elastic moduli of EVA and E-TPU/EVA membranes. This phenomenon is considered to be as a result of the influence of intermolecular forces and chain entanglements. For the TPU film, the EVA film and the E-TPU/EVA film, the application of an external force tended to unravel chain entanglements resulting in the elastic modulus being less at strains below 300%. However, the elastic modulus of the E-TPU membrane increased with an increase in strain to 100%. This is because the intermolecular forces rose as the stretch ratios increased due to the reorientation of molecular chains. This behavior predominated over the effect of the disentanglement and the breakage of fibers on the decline in elastic modulus. The situation reversed at strains above 100% and consequently led to a decrease in elastic modulus. Compared with pure TPU, the E-TPU membranes exhibited lower elastic moduli in the region of 0.10 MPa at strains of 5% which was similar to Young’s modulus for the linear proportion of the strain-stress curves. This was mainly due to the formation of porous structures in the electrospun membranes. By coating E-TPU with EVA, the elastic moduli of E-TPU/EVA membranes were obviously reduced when compared with pure TPU samples. In compliance with Equation (1), the actuated strain of DEs increased with decreasing Young’s modulus; hence it was expected that the E-TPU/EVA membranes would have excellent electromechanical properties.

**Figure 6:**
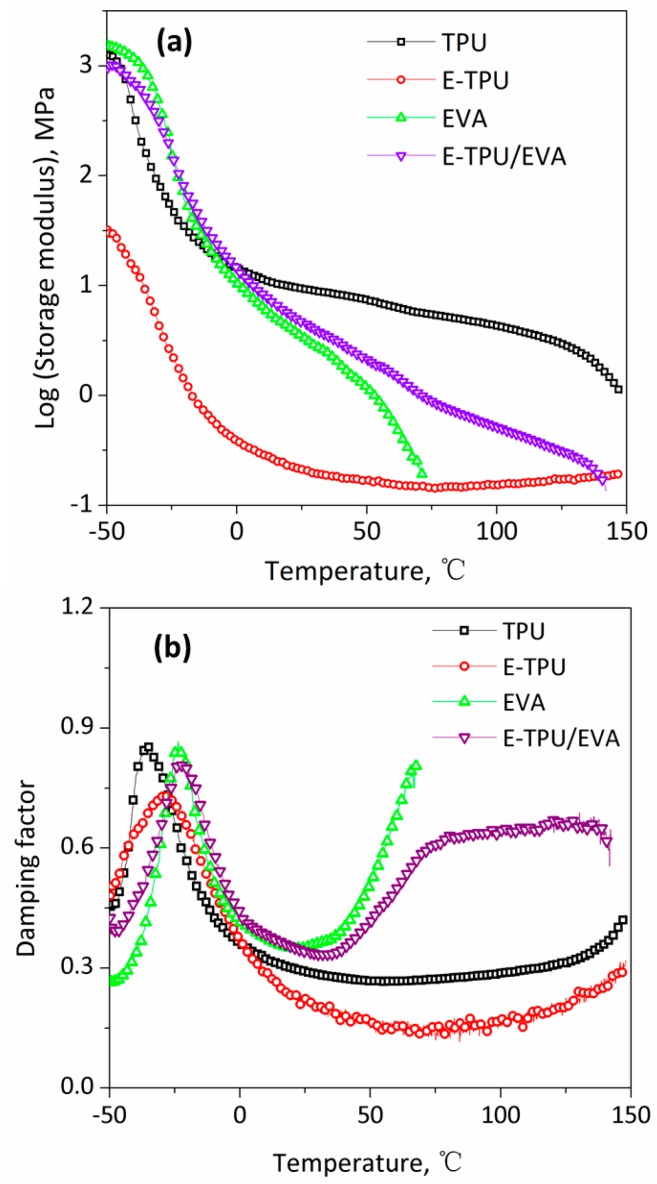
The dependence of (**a**) storage modulus and (**b**) damping factor on temperature.

DMA analysis is often used to determine a material’s storage modulus and damping factor. The dynamic storage modulus is similar to the Young’s or elastic modulus while the damping factor provides insight into the relative contributions of the viscous and elastic components of a viscoelastic material [37]. The storage modulus and damping factor of TPU, E-TPU, EVA and E-TPU/EVA membranes plotted against temperature are shown in Figure 6. It can be seen from Figure 6a that the storage modulus decreased with an increase in temperature for each sample, and this was because under the glass transition temperature, the free volume increases with heating which allows more side chain movement and above the glass transition temperature, the molecular main chains begin to move with improved mobility at elevated temperatures. The storage modulus of E-TPU was the lowest among all samples and varied slightly for the whole range of temperatures. This was probably due to the formation of a porous structure in the E-TPU sample [38]. No DMA data were collected for the EVA film above 72 °C and the E-TPU/EVA film above 142 °C due to their melting. This revealed that the TPU had a wider working temperature range than EVA. Figure 6b shows the change of damping factor with increasing temperature for TPU, E-TPU, EVA and E-TPU/EVA samples. It can be observed that the glass transition temperature of pure TPU film was about −36 °C which was the lowest among all samples. However, for E-TPU membranes, the glass transition temperature was slightly higher at −29 °C. This was because the stretch produced by electrostatic force during the electrospinning process contributed to the enhancement of the degree of crystallinity of the TPU which consequently led to an increase in glass transition temperature [39]. The EVA and E-TPU/EVA had a similar glass transition temperature of −23 °C. At a temperature below 0 °C, the damping factor of EVA was higher than that of TPU and E-TPU/EVA while the damping factor of TPU became a maximum as the temperature climbed above 0 °C. It is worth noting that the melt point of TPU was the highest which indicated that the thermal stability of TPU was superior to that of EVA and E-TPU/EVA over a temperature range of 0 to 150 °C.

**Figure 7:**
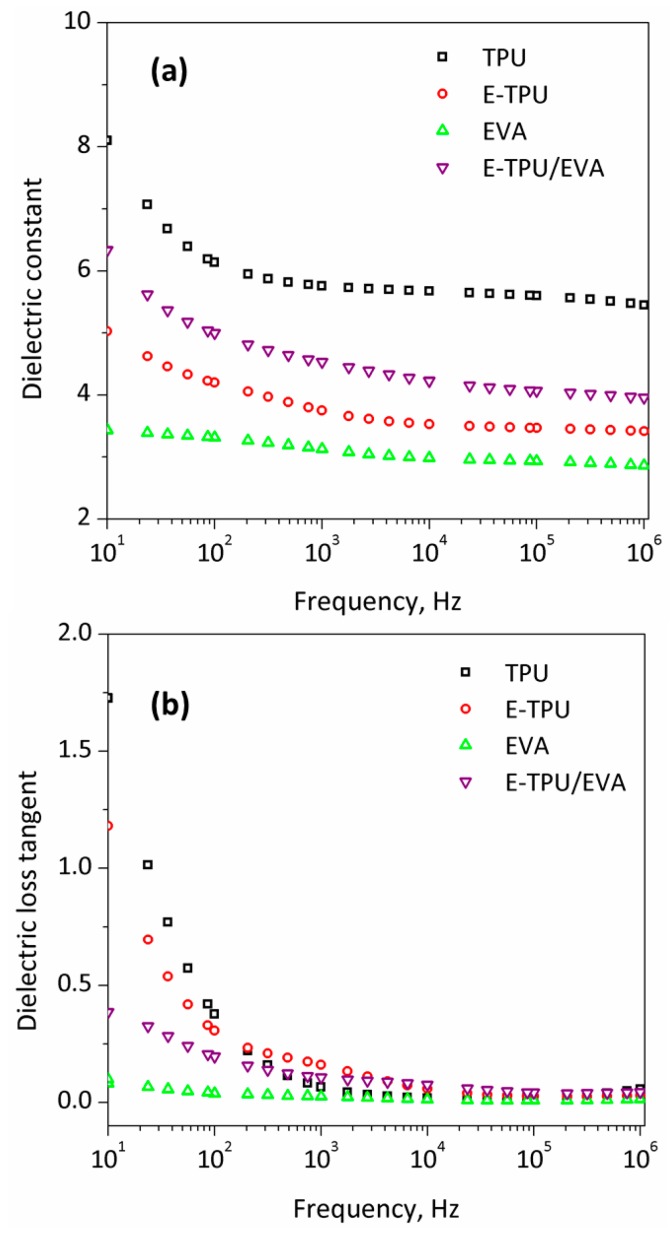
Dielectric spectra of TPU, electrospun TPU (E-TPU), ethylene vinyl acetate copolymer (EVA) and E-TPU/EVA: (**a**) dielectric constant related to frequency (**b**) dielectric loss tangent related to frequency.

The dielectric properties of the samples were determined by using dielectric spectroscopy. As can be seen from Figure 7a, the dielectric constant decreased with increasing frequency across the entire frequency range, indicating the presence of Maxwell polarization for all samples [40]. TPU possessed the highest dielectric constant of 5.7 at 1000 Hz due to its highest molecular polarizability. For the E-TPU fiber membrane, the dielectric constant dropped to approximately 3.7 because of its porosity [41]. The dielectric constant of EVA film was the lowest at about 3.1 and remained almost constant for the whole frequency range. Actually, EVA comprises random copolymers composed of vinyl acetate polar units and ethylene apolar units. The interchain dipolar interactions are expected to decrease as a consequence of the decreasing vinyl acetate content [42]. By coating EVA on an E-TPU fiber membrane, pores were nearly eliminated as shown in Figure 3d. Thus, the EVA/E-TPU composite presented an enhanced dielectric constant of about 4.5, which was higher than that of both E-TPU and EVA. The relation between dielectric loss tangent and frequency for all samples is shown in Figure 7b. In the lower frequency region, there was a more significant dielectric loss due to the loss associated with ionic mobility [43]. The high dielectric loss tangent occurred in all samples except the EVA film whose functional groups mainly comprised amide groups with high polarizability in TPU.

**Figure 8:**
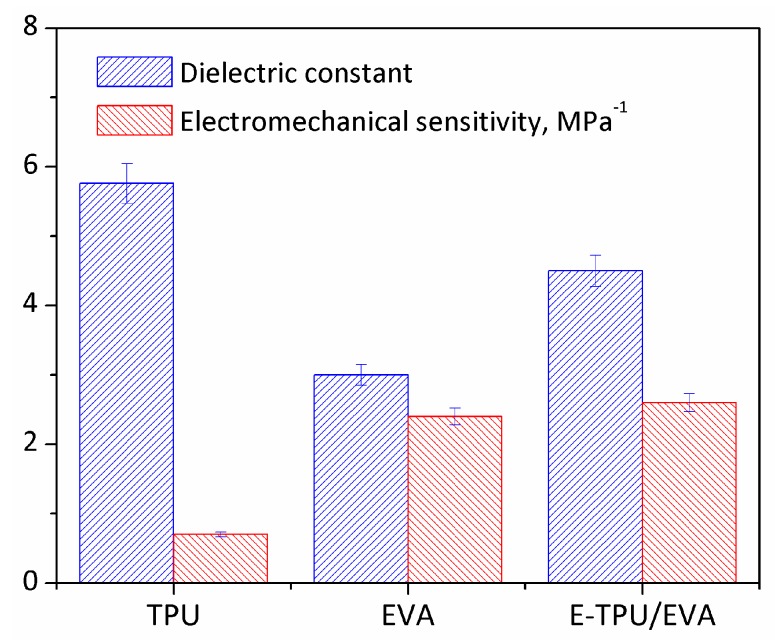
A chart of dielectric constant and electromechanical sensitivity for the three materials.

The electromechanical sensitivity, which is defined as the ratio of dielectric constant to Young’s modulus, was considered as a significant parameter in determining the voltage-induced strain of DEs [44]. Figure 8 shows the plots of dielectric constant and electromechanical sensitivity for TPU membranes, EVA membranes and E-TPU/EVA membranes. It can be observed that pure TPU film presented the highest dielectric constant, but showed unexpectedly the lowest electromechanical sensitivity. This was due to its relatively higher stiffness compared with EVA and E-TPU/EVA. Among all samples, E-TPU/EVA exhibited the highest electromechanical sensitivity, which implies that E-TPU/EVA might achieve the largest voltage induced deformation in accordance with Equation (1).

**Figure 9:**
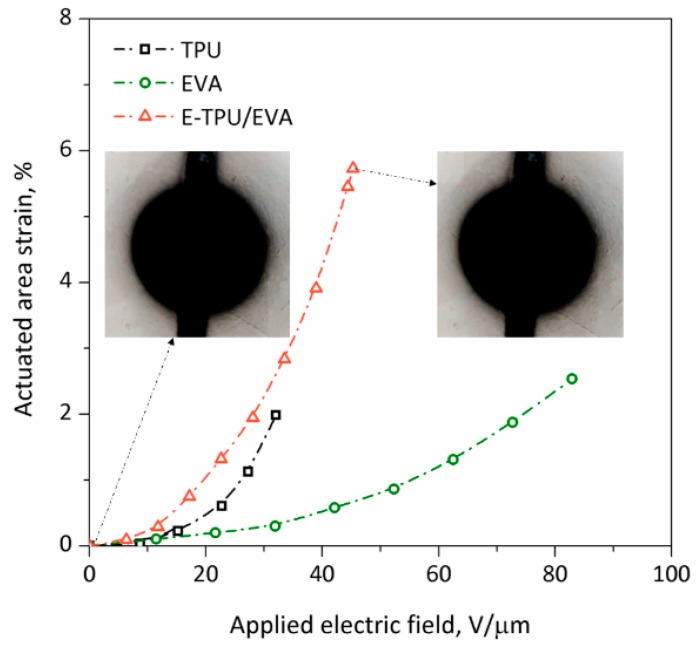
The actuated area strain of TPU, EVA and E-TPU/EVA membranes related to applied electric field.

The dependence of actuated area strain on applied electric field for pure TPU, pure EVA and E-TPU/EVA films was investigated to confirm these findings and the results are depicted in Figure 9. It was shown that the pure TPU film exhibited the minimum area strain of about 2% with an applied electric field of 32 V/μm, while EVA displayed a higher voltage-induced deformation of approximately 2.5% at a maximum electric field of 82 V/μm. Among all the materials tested, E-TPU/EVA achieved the highest voltage-induced deformation of above 6% in the absence of pre-stretch, which was 3 times larger than that of pure TPU film at a lower electric field of about 45 V/μm. It is known that the mechanism of electric breakdown for insulated polymers was elucidated as the consequence of the formation of cavities or low density regions in the bulk of the polymers [45]. The external pressure, which undoubtedly hinders formation of the regions with reduced density, increased the electrical strength of polymers [46]. Thus, the pre-stretch of fibrous TPU network retained by EVA greatly improved the electromechanical properties by enhancing electric breakdown strength as previously reported [47].

## 4. Conclusions

A novel DE material was fabricated by combining a soft electrospun TPU membrane with EVA. The pre-stretched TPU fibers embedded in EVA presented a muscle-like structure and possessed excellent electromechanical properties. Quasi-static and dynamic mechanical tests proved that the Young’s modulus and tangent modulus of E-TPU was lower than those of pure TPU, which is beneficial for electromechanical performance. The dielectric constant of the E-TPU membrane was enhanced by incorporating EVA. The fabricated E-TPU/EVA membrane also possessed enhanced electromechanical sensitivity when compared with EVA and TPU films, and this consequently led to large voltage induced deformations of above 6% for E-TPU/EVA, which was approximately 3 times larger than that of pure TPU film. Overall, the current study provides a description of an easy and efficient way to fabricate muscle-like soft DE materials with excellent electromechanical properties. These materials have great potential in for use in important DE applications. Future work will address the improvement of interfacial properties between EVA and E-TPU during the coating process which will further lead to the manufacture of DE composites with improved properties.

## Figures and Tables

**Table 1:** The tensile property data for different samples.

Polymer	Tensile Strength, MPa	Elongation at Break, %	Elastic Modulus, MPa (At a Strain of 5%)
TPU	8.9	1000	8.17
E-TPU	0.9	300	0.10
EVA	3.0	1500	1.35
E-TPU/EVA	2.4	1100	1.50
